# Obstructive sleep apnea in postmenopausal women: a comparative study using drug induced sleep endoscopy^☆^

**DOI:** 10.1016/j.bjorl.2016.03.011

**Published:** 2016-04-25

**Authors:** Soo Kweon Koo, Gun Young Ahn, Jang Won Choi, Young Jun Kim, Sung Hoon Jung, Ji Seung Moon, Young Il Lee

**Affiliations:** a Busan Saint Mary's Hospital, Department of Otorhinolaryngology, Busan, South Korea; b Ulsan Hana ENT Hospital, Ulsan, South Korea; c Good Samsun Hospital, Department of Occupational and Environmental Medicine, Busan, South Korea

**Keywords:** Sleep apnea syndrome, Endoscopy, Gender, Menopause, Síndrome da apneia do sono, Endoscopia, Gênero, Menopausa

## Abstract

**Introduction:**

The key to successful treatment of OSAS is to individually tailor such treatment. Thus, it is very important to determine the severity of OSAS, its pattern, and the extent of collapse, by gender, age, and BMI.

**Objective:**

The objective of the study was to understand the characteristics of obstructive sleep apnea in postmenopausal women by comparing postmenopausal and premenopausal subjects, and men, using DISE. We hope that our work will help the medical community to consult on, diagnose, and treat OSAS more effectively.

**Methods:**

A total of 273 patients (195 males and 78 females) diagnosed with OSAS were enrolled. Female patients were divided into pre-menopausal (*n* = 41) and post-menopausal patients (*n* = 37). The group of post-menopausal female patients was matched with a group of male patients with similar age and body mass index (BMI). DISE findings were compared between pre-menopausal female patients and post-menopausal female patients, and also between post-menopausal female patients and male patients matched for age and BMI.

**Results:**

Upon PSG examination, post-menopausal patients (who had a significantly higher BMI than did pre-menopausal patients; 25.6 kg/m^2^ vs. 23.5 kg/m^2^; *p* = 0.019) tended to have a higher AHI and a lower lowest SaO_2_, but the differences did not attain statistical significance. With DISE analysis, post-menopausal female patients showed higher values in all obstruction sites, with significantly higher value in lateral diameter of retropalatal (1.49 vs. 0.90; *p* = 0.001) and retrolingual levels (1.14 vs. 0.61; *p* = 0.003) compared to pre-menopausal females patients. Post-menopausal female patients showed significantly more retrolingual collapse (antero-posterior, AP, *p* ≤ 0.0001, and lateral diameter, *p* = 0.042) in the lower BMI group (BMI < 25) and more concentric retropalatal collapse (lateral diameter, *p* = 0.017 and tonsillar obstruction, *p* = 0.003) in higher BMI group (BMI ≥ 25) than BMI and age matched male patients.

**Conclusion:**

Post-menopausal female patients showed a different pattern of airway obstruction compared to pre-menopausal female patients and male patients matched for age and BMI based on DISE findings.

## Resumo

**Introdução:**

A chave para o sucesso do tratamento da síndrome da apneia obstrutiva do sono (SAOS) é adaptar individualmente esse tratamento. Assim, é muito importante determinar a gravidade da SAOS, seu padrão e a medida do colapso, por sexo, idade e IMC.

**Objetivo:**

O objetivo do estudo foi compreender as características da apneia obstrutiva do sono em mulheres na pós-menopausa, comparando estas características entre mulheres na pós-menopausa e pré-menopausa, e homens, utilizando endoscopia do sono induzido por fármacos (DISE). Esperamos que o nosso estudo ajude a comunidade médica a diagnosticar e tratar a SAOS de maneira mais eficaz.

**Método:**

Foram recrutados 273 pacientes (195 do sexo masculino e 78 do feminino) com diagnóstico de SAOS. As pacientes do sexo feminino foram divididas em pacientes na pré-menopausa (n = 41) e na pós-menopausa (n = 37). O grupo de pacientes do sexo feminino na pós-menopausa foi pareada com um grupo de pacientes do sexo masculino com idade e Índice de Massa Corporal (IMC) semelhantes. Os achados da DISE foram comparados entre as pacientes do sexo feminino na pré-menopausa e as pacientes do sexo feminino pós-menopausa e também entre pacientes do sexo feminino na pós-menopausa e pacientes do sexo masculino pareados por idade e IMC.

**Resultados:**

Ao exame de PSG, as pacientes na pós-menopausa (que tinham um IMC significativamente maior do que as pacientes na pré-menopausa; 25,6 vs 23,5 kg/m^2^; *p* = 0,019) tenderam a ter um IAH superior e uma saturação arterial de oxigênio (SaO_2_) mínima menor, mas as diferenças não atingiram significância estatística. Na análise do DISE, pacientes do sexo feminino pós-menopausa apresentaram valores mais elevados em todos os locais de obstrução, com um valor significativamente maior de diâmetro lateral dos níveis retropalatal (1,49 *vs.* 0,90; *p* = 0,001) e retrolingual (1,14 *vs.* 0,61; *p* = 0,003) em comparação com pacientes do sexo feminino na pré-menopausa. As pacientes do sexo feminino na pós-menopausa apresentaram colapso significativamente mais retrolingual (anteroposterior, AP, *p* ≤ 0,0001 e diâmetro lateral, *p* = 0,042) no grupo de IMC menor (IMC < 25) e colapso retropalatal mais concêntrico (diâmetro lateral, *p* = 0,017 e obstrução tonsilar, *p* = 0,003) no grupo de maior IMC (IMC ≥ 25) do que pacientes do sexo masculino pareados por IMC e idade.

**Conclusão:**

Com base nos achados do DISE, as pacientes do sexo feminino na pós-menopausa apresentaram um padrão diferente de obstrução das vias respiratórias em comparação com pacientes do sexo feminino na pré-menopausa e com os pacientes do sexo masculino pareados por idade e IMC.

## Introduction

Several population based studies across various geographical regions and ethnic groups have reported the prevalence of sleep disordered breathing (SDB) from obstructive sleep apnea syndrome (OSAS). OSAS is a disease that is relatively more common in men than women, especially middle-aged men.^1^ And according to various studies, incidence of OSAS in women increases after menopause.^1^, ^2^ Thus besides gender differences, incidence of OSAS may be due to the influence of hormones changing with age, racial makeup and differences in diet.

Treatment options for OSAS include weight management, use of continuous positive airway pressure (CPAP) machine, and surgical treatment; however, the key to success is in having a personalized management plan. In this respect, according to gender and age, determination of the severity of OSAS, pattern and degree of airway collapse are very important in individually tailoring a treatment. A comparative study of OSAS between men and women based on the natural physiologic sleep state of the subjects has not been conducted yet. Sleep nasoendoscopy or drug induced sleep endoscopy (DISE), first introduced by Croft and Pringle^3^ in 1991, is a procedure that uses a flexible nasoendoscope to visualize the upper airway under sedation. DISE evaluates the localization of flutter and collapse in patients with SDB. Several centers are now available that perform DISE is now available in multiple sleep study clinics. Although DISE has some limitations such as limited period of observation and multiple classification systems, it addresses the natural physiologic state of sleep more than any other currently available diagnostic tool.^4^

The key to successful treatment of OSAS is to individually tailor such treatment. Thus, it is very important to determine the severity of OSAS, its pattern, and the extent of collapse, by gender, age, and BMI; then it is possible to construct a personalized treatment plan. Menopause is associated with important physical and hormonal changes in females, and thus must be considered when therapeutic approaches are planned. We sought to understand the characteristics of obstructive sleep apnea in postmenopausal women by comparing postmenopausal and premenopausal subjects, and men, using DISE. We hope that our work will help the medical community to consult on, diagnose, and treat OSAS more effectively.

## Methods

### Patients selection and outcome assessment

The retrospective observational study was conducted at our ENT Department where DISE was performed by one examiner in 273 subjects (195 males, mean age 41.1 years old; and 78 females, mean age 46.0 years old) consecutive OSAS patients from 2013 to 2014.

Before DISE, all patients received a thorough ear, nose, and throat examination, focusing on nasal pathology, tonsil size, uvula and palate aspect and tongue size. Their medical history was taken. All patients underwent full night polysomnography (PSG, WEE-1000K, Nihon Kohden, Japan). To minimize morphological bias, we excluded patients with skeletal framework anomalies such as severe retrognathia or jaw retrusion patients. In addition, to exclude bias introduced by tonsil size, we excluded patients with tonsils of grades III and IV. Patients who had undergone prior surgery of the soft palate or tongue were also excluded. Post-menopause was defined as a patient who did not have menstruation for more than a year and post-menopausal women receiving hormonal replacement therapy were also excluded. The female groups consisted of 41 pre-menopausal (mean age, 36.7 years old) and 37 post-menopausal female patients (mean age, 56.3 years old). All post-menopausal female patients were over 50 years old. Individuals with a BMI of 25–29.9 were considered overweight, while individuals with a BMI 30 were categorized as obese.^5^ The matching limits were 50 years or older for age, and 25 kg/m^2^ or higher for BMI in our study.

According to these criteria, we compared DISE findings between pre-menopausal female and post-menopausal female patients or between post-menopausal female and males patients matched for age (over 50 years old) and BMI.

### Drug induced sleep endoscopy and classification system

All DISE procedures were performed by the same ENT surgeon in a semi-dark and silent operation room with each patient lying supine. Sleep was induced by intravenous administration of midazolam with respiratory monitoring. The anesthesiologist slowly titrated the drug to 0.07 mg/kg per patient; boluses of 1–2.5 mg were given (to a maximum of 7.5 mg per patient) using a target-controlled infusion system. An extra bolus was often required by patients who were extremely nervous. Once the patient was deeply asleep enough to snore, show obstructions and responded sluggish to a light glabella or loud auditory stimulus (Ramsay's level of sedation scale 5),^6^ a 4 mm-diameter flexible video laryngoscope was gently introduced through the nose. Video images of all DISE procedures were later evaluated by a single otolaryngologist.

We often performed DISE postoperatively to observe the extent of improvement after surgery; preoperative procedures were principally diagnostic in nature.

Each procedure took 20–30 min. When possible, we performed only one cycle, after careful preparation, to minimize the risk of respiratory complications.

We divided the pharynx into two portions: the retropalatal level (the region of posterior to the soft palate) and the retrolingual level (the region of the pharynx posterior to the vertical portion of the tongue). We made the classification system according to our published procedures.^4^, ^7^ Briefly, our classification system included the following: the obstruction site, degree of SDB, and the anatomical structure contributing the most to sleep apnea. The retropalatal level was subdivided into palate (antero-posterior diameter), lateral pharyngeal wall (lateral diameter), and tonsil (specific structure contributing to obstruction). The retrolingual level was divided into tongue base (antero-posterior diameter), lateral pharyngeal wall (lateral diameter), and epiglottis (specific structure contributing to obstruction). Degree of airway obstruction was categorized as no obstruction (rated as 0), partial obstruction (rated as 1; 50–75% obstruction), and complete obstruction (rated as 2, >75%) (Table 1).

**Table 1:** DISE classification system.

Obstruction level	Configuration^a^		
	AP diameter	Lateral diameter	Contributing structure
Retropalatal	Palate AP	LPW	Tonsil
	0/1/2	0/1/2	0/1/2
Retrolingual	Tongue base AP	LPW	Epiglottis
	0/1/2	0/1/2	0/1/2

AP, anteroposterior.

a Degree of obstruction has one number for each structure: 0, no obstruction(no vibration); 1, partial obstruction (vibration, 50–75%); 2, complete obstruction (collapse, >75%).

### Statistical analysis

We evaluated the difference between female and male patient groups according to DISE findings. Student's *t*-test was used to compare BMI, AHI, and lowest arterial oxygen saturation (SaO_2_) between male and female patients. Mann–Whitney *U* test was used if the data was not normally distributed. All statistical tests were performed using SPSS version 18.0 (SPSS Inc., Chicago IL, USA). Statistical significance was met when *p*-value was less than 0.05.

The research protocol was reviewed and approved by the Institutional Review Board (IRB approval number BSM 2014-131).

## Results

Demography of patients used in this study is summarized in Table 2. Total 273 subjects (195 males, mean age 41.1 years old; and 78 females, mean age 46.0 years old). Male patients had significantly higher BMI (26.0 vs. 24.5; *p* = 0.001), higher AHI (23.2 vs. 13.9; *p* = 0.001), and lower lowest SaO_2_ (79.6 vs. 83.2; *p* = 0.026). The average ages of pre-menopausal and post-menopausal female patients were 36.7 and 56.3 years, respectively. Upon PSG examination, post-menopausal patients (who had a significantly higher BMI than did pre-menopausal patients; 25.6 kg/m^2^ vs. 23.5 kg/m^2^; *p* = 0.019) tended to have a higher AHI and a lower lowest SaO2, but the differences did not attain statistical significance (Table 2).

**Table 2:** Demography and patient's characteristics of PSG finding.

Sex	Number	Age (yr)	BMI (kg/m^2^)	AHI (events/h)	Lowest SaO_2_ (%)
*Male*	195	41.1	26.0	23.2	79.6
*Female*	78	46.0	24.5	13.9	83.2
*p*^a^		0.005^c^	0.001^c^	0.001^c^	0.026^c^

*Female*					
Pre-menopausal female patients	41	36.7	23.5	12.8	85.0
Post-menopausal female patients	37	56.3	25.6	15.0	81.2

*p*^b^		<0.0001^c^	0.019^c^	0.630	0.130

PSG, polysomnography; BMI, body mass index; AHI, apnea/hypopnea index; SaO_2_, arterial oxygen saturation.

a Student's *t*-test between male and female.

b Student's *t*-test between pre-menopausal and post-menopausal female patients.

c Statistically significant differences between groups regarding age, BMI, AHI and lowest SaO_2_.

Comparisons between pre-menopausal and post-menopausal female patients according to DISE finding are summarized in Table 3. In DISE finding, post-menopausal female patients showed higher values in all the obstruction sites. Significant difference was seen for both lateral diameter of retropalatal (0.90 vs. 1.49; *p* = 0.001) and retrolingual level measures (0.61 vs. 1.14; *p* = 0.003) (Table 3).

**Table 3:** Comparison of DISE score between pre-menopausal and post-menopausal female patients.

	Retropalatal level			Retrolingual level		
	AP diameter	Lateral diameter	Tonsillar obstruction	AP diameter	Lateral diameter	Epiglottic obstruction
Pre-menopausal female patients (41)	1.73	0.90	0.49	1.49	0.61	0.39
Post-menopausal female patients (37)	1.76	1.49	0.59	1.62	1.14	0.62
*p*^a^	0.847	0.001^b^	0.568	0.424	0.003^b^	0.227

(), number of patients; DISE, drug-induced sleep endoscopy; AP, anteroposterior.

a Student's *t*-test.

b Statistically significant differences between groups.

In comparison to DISE finding between post-menopausal female patients (*n* = 37) and male patients matched for age (over 50 years old) and BMI, the total number of male patients was 195, of these, 26 and 31 were matched for age (over 50 years) and BMI (BMI < 25 kg/m^2^), and age (over 50 years) and BMI (BMI ≥ 25 kg/m^2^), with females. Post-menopausal female patients had significantly higher values in AP (*p* < 0.0001) and lateral diameter of retrolingual level (*p* = 0.042) in lower BMI group (BMI < 25) (Table 4). In higher BMI group (BMI ≥ 25), post-menopausal female patients had significantly higher values in lateral diameter (*p* = 0.017) and tonsillar obstruction (*p* = 0.003) of retropalatal level (Table 5).

**Table 4:** Comparison of DISE score between post-menopausal female patients (BMI < 25) and men (age ≥ 50; BMI < 25).

	Retropalatal level			Retrolingual level		
	AP diameter	Lateral diameter	Tonsillar obstruction	AP diameter	Lateral diameter	Epiglottic obstruction
Men (26)	1.81	0.88	0.31	0.85	0.81	0.62
Post-menopausal female patients (17)	1.88	1.24	0.35	1.76	1.24	0.71
*p*^a^	0.710	0.166	0.935	<0.0001^b^	0.042^b^	0.716

(), number of patients; DISE, drug-induced sleep endoscopy; AP, anteroposterior.

a Mann–Whitney test.

b Statistically significant differences between groups.

**Table 5:** Comparison of DISE score between post-menopausal female patients (BMI ≥ 25) and men (age ≥ 50; BMI ≥ 25).

	Retropalatal level			Retrolingual level		
	AP diameter	Lateral diameter	Tonsillar obstruction	AP diameter	Lateral diameter	Epiglottic obstruction
Men (31)	1.68	1.19	0.16	1.10	0.87	0.52
Post-menopausal female patients (20)	1.65	1.70	0.80	1.50	1.05	0.55
*p*^a^	0.882	0.017^b^	0.003^b^	0.057	0.461	1.000

(), number of patients; DISE, drug-induced sleep endoscopy; AP, anteroposterior.

a Mann–Whitney test.

b Statistically significant differences between groups.

## Discussion

OSAS is generally more common and severe in men and than women when matched for BMI and PSG parameters such as AHI and lowest SaO_2_ saturation.^1^ These findings are in agreement with our result and previous reports in the literature about sleep apnea patients of different ethnic origins.^8^, ^9^, ^10^ However, women experience hormone-related changes such as menopause and pregnancy that have a great influence on incidence OSAS. Therefore, gender differences are very important in examination and treatment of sleep disorders.

OSAS generally occurs more frequently and with higher severity in post-menopausal females than pre-menopausal females as pre-menopausal females have higher levels of circulating progesterone that could prevent airway closure by reducing the collapsibility of genioglossus muscle.^10^, ^11^, ^12^, ^13^, ^14^, ^15^ Other than that the reasons behind influence of gender and the menopause status for females in occurrence and severity of OSAS are still not completely understood. It has been estimated that the prevalence of this condition among women in their sixth or seventh decade of life ranges from 4% to 22% depending on the definition used and the population examined.^11^ In our study, post-menopausal female patients with OSAS had a significantly higher value of BMI, higher AHI and lower lowest SaO^2^ saturation, although the differences were not statistically significant. Therefore, post-menopausal female patients tended to show more severe OSAS than pre-menopausal female patients. Although no clear causes for these changes have been established, the age parameters for menopausal status and upper airway muscle tone might have contributed.

In our study, post-menopausal female patients showed a tendency with a more severe airway obstruction for all obstruction sites, with significantly higher value in both lateral diameter of retropalatal and retrolingual level compared to pre-menopausal female patients. This suggests that the lateral wall of the airway obstruction plays a significant role for women after menopause. This is an important point from the therapeutic point of view. Other research studies from DISE finding of obstructive sleep apnea patients showed similar results, emphasizing the importance of the lateral diameter.^4^, ^16^, ^17^ In the current study from DISE analysis, multilevel and complete collapse was more prevalent in obese patients and in those with more severe OSA. Higher BMI values were associated a higher probability of complete concentric palatal collapse and also complete lateral hypopharyngeal collapse, which may be explained by fat accumulation at the lateral pharyngeal walls.^16^ On the contrary, lower BMI value was associated with tongue base collapse, and this may be result of less fat accumulation in the lateral pharyngeal wall, possibly allowing for more backward movement of the tongue.^16^ In our study, post-menopausal female patients showed more concentric palatal collapse (lateral diameter and tonsillar obstruction of retropalatal level) in higher BMI group and retrolingual collapse (AP and lateral diameter of retrolingual level) in lower BMI group than male patients, and our result is in agreement with previous reports. These findings indicate that post-menopausal female patients have a higher tendency of anatomical change in the airway than men of the same age group and level of obesity. Therefore, we should pay more attention to anatomical changes when treating post-menopausal female OSAS patients.

In general, a large upper airway soft tissue volume in men may contribute to increased prevalence of OSAS compared to women. Upper airway resistance is reported to be higher in men than that in women.^17^, ^18^ But, post-menopausal female patients showed more severe closure in our study. This might be due to the fact that the muscle tone of post-menopausal women is decreased. However, further research is required to explore this possibility.

Our study has some limitations. The study population was small thus reducing the power of analysis. Also the patients of this study were candidates for obstructive sleep apnea surgery, so did not represent the general OSAS population. As a result, there was an inherent bias for drug selection, procedure, and classification from DISE. A large-scale study of the general OSAS population is required, as it would obviate this treatment bias.

## Conclusion

In conclusion, post-menopausal female patients showed more severe sleep apnea than pre-menopausal female patients. In DISE findings, post-menopausal female patients showed a tendency with a more severe airway obstruction, especially in the lateral wall. Post-menopausal female patients showed more concentric palatal collapse in higher BMI group and retrolingual collapse in lower BMI group than the male patients. Post-menopausal female patients showed a tendency with more severe airway obstruction in all parts of the airway in DISE finding than men with similar age and BMI. The key to successfully treating OSAS is individually tailored treatment. In this respect, determining the severity of OSAS, pattern and degree of collapse according to gender, age and BMI are very important for a personalized treatment plan. Therefore, within some limits, our comparative study between men and women through DISE may help the medical community consult, diagnose, and treat OSAS more effectively.

## Conflicts of interest

The authors declare no conflicts of interest.
